# Broadband polarization conversion with anisotropic plasmonic metasurfaces

**DOI:** 10.1038/s41598-017-09476-8

**Published:** 2017-08-18

**Authors:** Wei Cao, Xiaodong Yang, Jie Gao

**Affiliations:** Department of Mechanical and Aerospace Engineering, Missouri University of Science and Technology, Rolla, MO 65409 USA

## Abstract

Metasurfaces offer exciting opportunities that enable precise control of light propagation, optical intensity, phase and polarization. Plasmonic metasurface based quarter-wave plates have been recently studied to realize the conversion between linear polarization and circular polarization. However, it is still quite challenging to directly measure the birefringent phase retardation introduced by metasurface wave plates with a reliable technique. Here, we report a high-performance broadband metasurface quarter-wave plate made of anisotropic T-shaped plasmonic antennas in near-infrared wavelength range, where the achromatic nearly 90° transmitted phase retardation through the metasurface is precisely characterized with an optical vortex based interferometric approach. Based on the measured transmission amplitude and phase of two orthogonal linear polarization components, nearly unit degree of linear polarization is extracted from the Stokes parameters, indicating excellent broadband polarization conversion between linearly and circularly polarized light through the metasurface. Our results will be an important step forward in the advancement of integrated metasurface devices for polarization conversion and beam manipulation, structured light control, as well as new spectroscopic and interferometric techniques for metasurface characterization.

## Introduction

Polarization is one of the significant parameters to describe the properties of light. The manipulation of the polarization state of light has played a key role in a wide range of applications^1–3^. It is well known that a quarter-wave plate enables the invertible polarization conversion between linearly and circularly polarized light due to the equal transmitted amplitude and a 90° phase retardation between two orthogonal linear polarization components. A bulk birefringent crystal is conventionally employed to achieve the required phase retardation between the two orthogonal components of o-ray and e-ray, where the phase delay depends on both the birefringence and thickness of crystal. Since the birefringence of commonly available crystals is weak, conventional wave plates usually have large physical thickness in order to obtain the desired phase retardation, which greatly hinders the miniaturization of bulky wave plates and restricts the development of on-chip optics and photonics integration. Such limitation has been recently overcome by adopting ultracompact wave plate design with metamaterials and metasurfaces. Metamaterials and metasurfaces are artificial, subwavelength structured media capable of realizing unconventional electromagnetic properties not existing in nature and providing unique platforms for the demonstration of exotic functionalities, such as abrupt phase or polarization change, near-zero permittivity, extraordinary anisotropy, cloaking, negative refraction, perfect absorption, modulation, and sensing^4–14^. Ultrathin metasurfaces have been designed to demonstrate versatile applications in optical vortex beam generation, flat lenses, spin-hall effect of light, and holographic imaging^15–21^. Recently, metamaterial or metasurface based wave plates used for polarization manipulation have been proposed^22–32^, where great efforts are devoted to realizing broadband polarization conversion devices. The anisotropic optical resonances along two orthogonal directions are introduced in designing metamaterial or metasurface based wave plates in order to generate the desired phase retardation^22, 28, 29, 32^. In addition, metasurfaces made of phased antenna arrays are also demonstrated to generate scattered light with arbitrary polarization states^24^. Metamaterial or metasurface based wave plates have provided a promising pathway towards the effective polarization control while keeping device size compact.

The phase shift induced by the optically thin metamaterial or metasurface nanostructures is one critical parameter to describe the optical properties of the designed wave plates. However, it is still quite challenging to directly measure the phase shift through the metamaterial or metasurface under a certain polarization and the birefringent phase retardation introduced by the metamaterial or metasurface based wave plates with a reliable technique. Although conventional interferometry or spectroscopic ellipsometry have been considered to characterize the phase shift from metamaterials^33–40^, these ordinary techniques are still complicated or inapplicable for metamaterial characterization. There is one recently proposed interferometric approach based on optical vortex with a helical wavefront that offers a promising solution to directly visualize the phase shift from metamaterials^41^, by observing the rotation of spiral interference pattern according to the vortex based interferometry^42–44^.

In this work, we propose and demonstrate a new design of metasurface quarter-wave plate made of anisotropic T-shaped plasmonic antenna array covering a broadband near-infrared wavelength range. The optical vortex based interferometric approach is utilized to directly measure the transmitted achromatic nearly 90° phase retardation through the metasurface between two orthogonal linear polarization components. We further analyze the optical transmission amplitude and phase spectra together with the plasmonic resonant modes of the T-shaped antenna under linear polarization along two orthogonal antenna arms, indicating that the desired equal transmission amplitude and nearly 90° birefringent phase retardation between two orthogonal directions for realizing a quarter-wave plate are tailored by properly tuning the anisotropic plasmonic dipolar resonances in the two arms of T-shaped antenna. Based on the simulated and measured transmission amplitude and phase retardation from the metasurface, nearly unit degree of linear polarization is extracted from the Stokes parameters across a wide near-infrared wavelength range, demonstrating the high-performance broadband linear-to-circular polarization conversion through our currently designed metasurface quarter-wave plate.

## Results

### Design of anisotropic T-shaped plasmonic antenna

**Figure 1:**
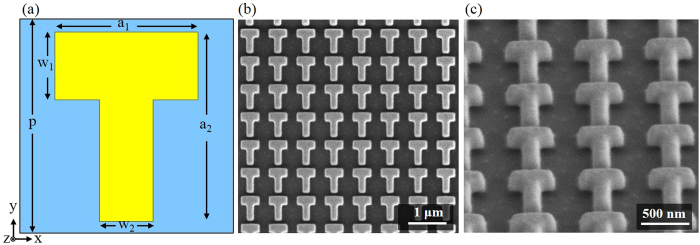
(**a**) Schematic of the designed T-shaped antenna unit cell and the geometric dimensions: *a*_1_ = 475 nm, *a*_2_ = 630 nm, *w*_1_ = 225 nm, *w*_2_ = 180, *p* = 720 nm, and the gold film thickness is 180 nm. (**b**) and (**c**) Top-view and perspective-view SEM images of the fabricated T-shaped antenna array on glass substrate.

The schematic of our designed T-shaped plasmonic antenna for building metasurface quarter-wave plate is shown in Fig. 1(a). The gold T-shaped antenna on silica substrate has horizontal and vertical arms of different lengths *a*_1_ and *a*_2_, and the widths of the two arms are *w*_1_ and *w*_2_, respectively. The period of the antenna unit cell is *p*. The lengths and widths of two antenna arms are varied to tune the anisotropic plasmonic dipolar resonances into the designed near-infrared wavelengths. Numerical simulations with CST Microwave Studio software are performed to design the geometric dimensions of the T-shaped antenna, where the refractive index of silica is 1.47 and the dielectric function of gold is from the Drude mode with plasmon frequency *ω*_*p*_ = 1.37 × 10^16^ rad/s and damping constant *γ*_*p*_ = 4.08 × 10^13^ rad/s. By considering the surface scattering and grain boundary effects in thin gold film, the damping constant is set as six times greater than that of the bulk gold^34, 45^, in order to match the experimental results. Boundary conditions of the unit cell are defined as open boundaries with perfectly matched layers along both *x* and *y* directions, and light incidence and propagation is along *z* direction. The designed metasurface is excited by linearly polarized plane wave along either *x* or *y* direction to the metasurface normal. The corresponding transmission amplitude and phase spectra of two orthogonal linear polarization components from the T-shaped antenna array are calculated. The final designed geometric dimensions of the T-shaped antenna unit cell are *a*_1_ = 475 nm, *a*_2_ = 630 nm, *w*_1_ = 225 nm, *w*_2_ = 180, *p* = 720 nm, and the gold film thickness is 180 nm. The designed metasurface is then fabricated in a 180 nm-thick gold layer deposited on a glass substrate using electron beam evaporation. A 3 nm-thick titanium layer is deposited between the gold layer and the glass substrate for adhesion purpose. The T-shaped antenna array is milled in the gold film using focus ion beam (FIB, FEI Helios Nanolab 600 Dual Beam system). The fabricated metasurface sample area is around 60 μm by 60 μm. Figure 1(b) and (c) show the scanning electron microscope (SEM) images of the top view and perspective view for the fabricated T-shaped antenna array. As the incident light is linearly polarized along the horizontal or vertical direction, the plasmonic dipolar resonance along one of the antenna arms will be excited. The quarter-wave plate functionality will be realized in the wavelength range between the two anisotropic plasmonic dipolar resonant modes, where both the equal amplitude and nearly 90° phase retardation along two orthogonal directions can be achieved simultaneously.

Each T-shaped antenna unit cell can be considered as a miniature quarter-wave plate. The general relation between the incident electric field *E* ^i^ and the transmitted electric field *E* ^o^ at normal incidence can be expressed with the transmission matrix (Jones matrix) as^22, 23^

1 $${E}^{o}=T{E}^{i}=[\begin{array}{cc}{t}_{xx} & {t}_{xy}\\ {t}_{yx} & {t}_{yy}\end{array}]\,[\begin{array}{c}{E}_{x}^{i}\\ {E}_{y}^{i}\end{array}]$$

where *t*_ij_ terms represent the transmitted complex amplitudes. This operation can be obtained without the cross-coupling terms so that *t*_xy_ = *t*_yx_ = 0 in a suitably chosen reference system.

2 $$[\begin{array}{c}{E}_{x}^{o}\\ {E}_{y}^{o}\end{array}]=\,[\begin{array}{cc}{t}_{xx} & 0\\ 0 & {t}_{yy}\end{array}]\,[\begin{array}{c}{E}_{x}^{i}\\ {E}_{y}^{i}\end{array}]$$

where the phase of the transmitted electric field along *x* and *y* directions can be expressed as

3 $${\varphi }_{xx}={\rm{\arg }}({t}_{xx})$$

4 $${\varphi }_{yy}={\rm{\arg }}({t}_{yy})$$

The transmission amplitude ratio between the two orthogonal directions is defined as *R* = |*t*_yy_|/|*t*_xx_|, and the phase retardation is Δ*ϕ* = *ϕ*_yy_ − *ϕ*_xx_. The complex amplitudes of transmitted field through a quarter-wave plate should satisfy both *R* = 1 and Δ*ϕ* = 90° at the same time. In our current design, the transmission amplitude and phase retardation for two orthogonal linear polarizations are tailored by varying the geometric dimensions of T-shaped antenna in order to meet such requirement. In order to achieve a quarter-wave plate in metasurfaces, it is key to realize a phase retardation Δ*ϕ* = *ϕ*_yy_ − *ϕ*_xx_ equal to 90° at the crossing point of transmission amplitude for the two orthogonal polarization components. It is quite helpful to modify the surface plasmon propagation along the arms of the two orthogonal polarization components in order to improve the phase retardation by properly increasing the thickness of T-shaped antennas. In addition, in order to improve the transmission amplitude at the crossing point of two orthogonal components and broaden the bandwidth over which the phase retardation is around 90°, one should properly adjust both the lengths of horizontal and vertical arms of T-shaped antennas. In our work, we properly detuned the two plasmonic resonances to obtain a broad bandwidth quarter-wave plate and improve the transmission amplitude at the crossing point of the two orthogonal polarization components by shortening the horizontal arm to 475 nm and lengthening the vertical arm to 630 nm.

### Phase measurement with optical vortex based spiral interferometry

**Figure 2:**
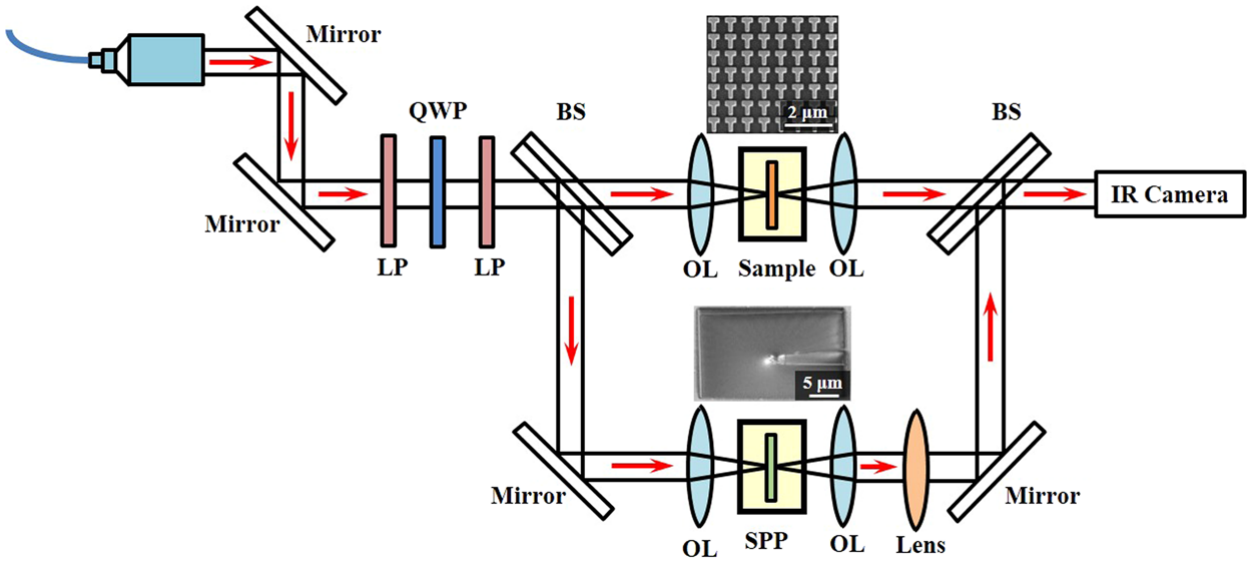
Experimental setup of the optical vortex based spiral interferometry for directly characterizing the phase retardation through the metasurface sample of T-shaped antenna array. LP: linear polarizer, QWP: quarter-wave plate, BS: beam splitter, OL: objective lens, SPP: spiral phase plate (with the perspective-view SEM image shown in the lower inset).

Figure 2 shows the experimental setup of the optical vortex based spiral interferometry for directly measuring the phase retardation under two orthogonal linear polarization excitations introduced by the metasurface sample. The optical beam is coupled with a fiber collimator from a tunable laser source covering wavelength from 1480 nm to 1580 nm. The generated linearly polarized laser beam is separated into two paths by a beam splitter. One beam is focused onto the metasurface sample at normal incidence, and the other beam is coupled via a glass spiral phase plate for creating an optical vortex beam with topological charge of one. The SEM image of the perspective view of the spiral phase plate fabricated on a glass slide using FIB milling is shown in the lower inset of Fig. 2. The transmitted beams are then interfered with each other to form a spiral interference pattern captured by an infrared camera. The phase shift of transmitted beam through the metasurface sample under certain linear polarization will be directly visualized by the rotation angle of the measured spiral interference pattern. Therefore, the phase retardation between two orthogonal linear polarizations from the metasurface sample can be obtained.

**Figure 3:**
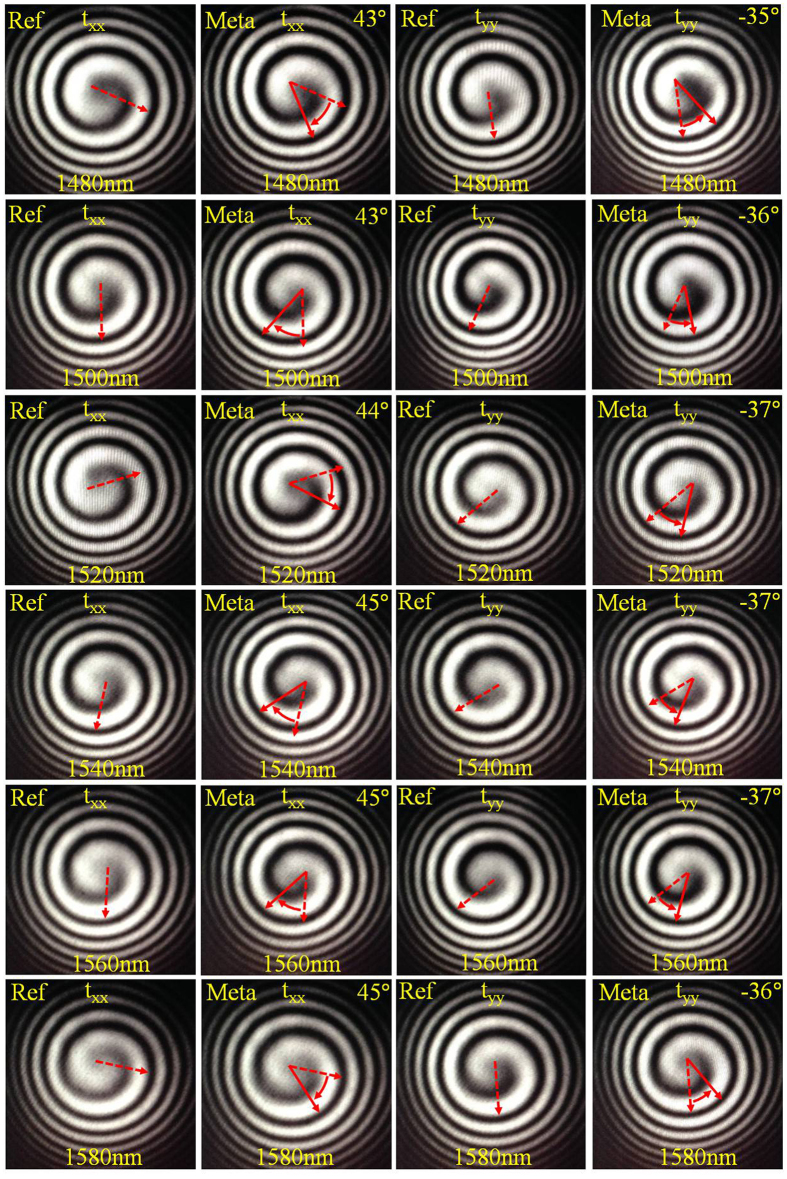
Recorded images of the vortex based spiral interference patterns for optical beam passing through the reference glass substrate (Ref) and the metasurface sample (Meta) under two orthogonal linear polarizations at wavelengths from 1480 nm to 1580 nm with a 20 nm interval. The rotation angles *ϕ*_*xx*_ and *ϕ*_*yy*_ of the spiral interference patterns from the metasurface relative to the reference glass substrate are listed.

In order to take into account the phase shift across the glass substrate, the optical vortex based spiral interference patterns are recorded for optical beam transmitted through both the reference glass substrate and the metasurface sample under two orthogonal linear polarizations at various wavelengths from 1480 nm to 1580 nm with a 20 nm interval, as displayed in Fig. 3 where the rotation angles of the spiral interference patterns from the metasurface sample relative to the reference glass substrate are measured. According to the previous studies^41, 42^, the equation describing the spiral interference pattern between a Gaussian beam and a vortex beam with topological charge of one can be written as *θ* = *ar* ^2^ + *b*_0_, where *r* and *θ* are the polar coordinates. *a* refers the rate at which the spiral rotates as its radius increases and it is directly related to the curvature of the interfering wavefront, while *b*_0_ is the starting phase of the spiral which shows the starting angle of spiral interference pattern at the center. For all the measured spiral interference patterns shown in Fig. 3, the curvature of the interfering wavefront does not change so that the parameter *a* can be considered as a constant. Therefore, the rotation angle of the spiral interference pattern is characterized by the parameter *b*_0_, which is directly related to the phase shift induced by the metasurface sample. The rotation angle is determined by analyzing the tangential direction at the center of the spiral based on first locating the minimum intensity in the spiral interference pattern^41, 42^. Then the relative phase shifts *ϕ*_xx_ and *ϕ*_yy_ under two orthogonal linear polarizations are obtained by comparing the rotation angles between the metasurface sample and the reference glass substrate. The phase retardation introduced by the metasurface is finally determined by Δ*ϕ* = *ϕ*_yy_ − *ϕ*_xx_. According to refs 41 and 42, the vortex based interferometric method can directly measure the phase changes of the transmitted light introduced by metasurfaces. We utilized this novel technique in measuring the phase retardation between two orthogonal linear polarizations at wavelengths from 1480 nm to 1580 nm with a 20 nm interval. A bare glass substrate was used as the reference for all sets of measurements. The transmitted beams under two orthogonal linear *x*-polarization and *y*-polarization through both the metasurface sample and glass substrate reference were characterized by using the vortex based interferometric method. The relative phase shift for *x*-polarization and *y*-polarization introduced by the metasurface can be firstly calculated by measuring the rotation angle and direction of the spiral interference pattern in comparison to that of glass substrate reference, respectively. For example, at the wavelength of 1540 nm in Fig. 3, the relative phase shift for *x*-polarization is 45° clockwise and the relative phase shift for *y*-polarization is 37° counterclockwise. It can be seen that the phase shift directions are opposite for *x*-polarization and *y*-polarization in terms of glass substrate reference. The rotation direction of spiral interference pattern is defined positive for clockwise and negative for counterclockwise. Thus, the phase retardation between two orthogonal linear *x*-polarization and *y*-polarization is calculated as 82°.

### Analysis of metasurface quarter-wave plate

**Figure 4:**
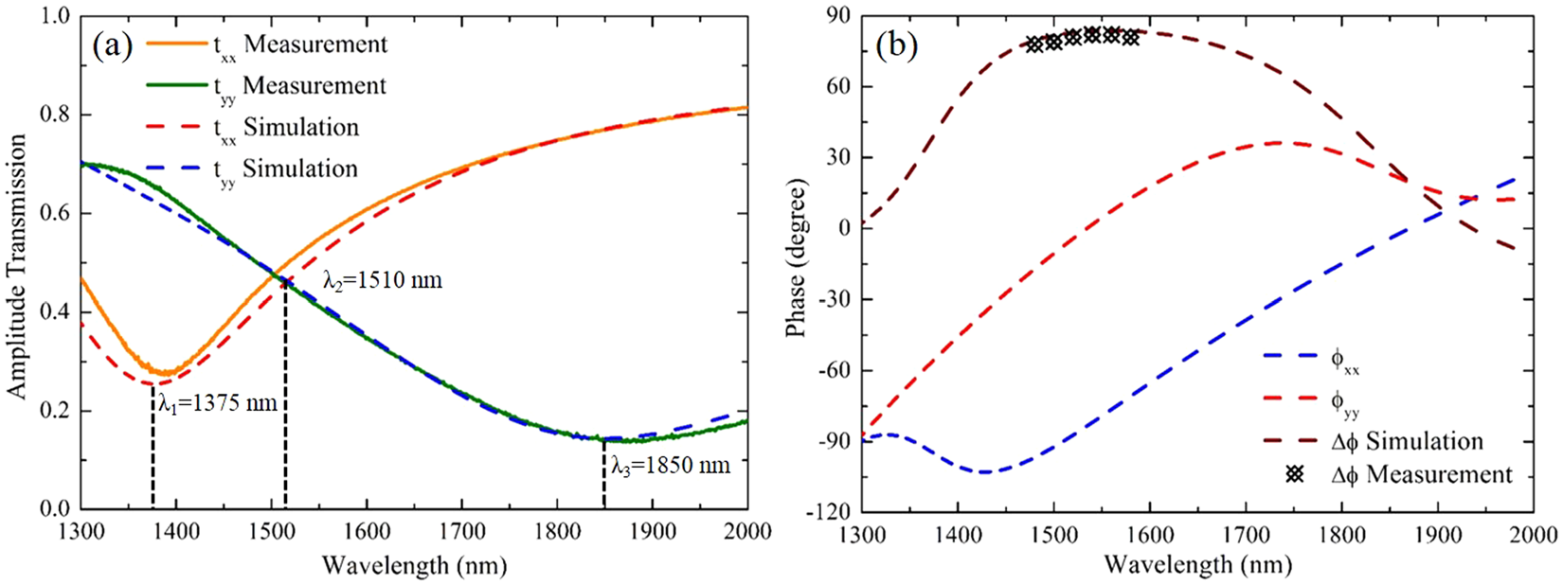
(**a**) Measured and simulated transmission amplitude spectra of *t*_xx_ and *t*_yy_ under two orthogonal linear polarization excitations along *x* and *y* directions. (**b**) Measured and simulated phase retardation between two orthogonal directions, together with simulated phase shifts *ϕ*_xx_ and *ϕ*_yy_.

**Figure 5:**
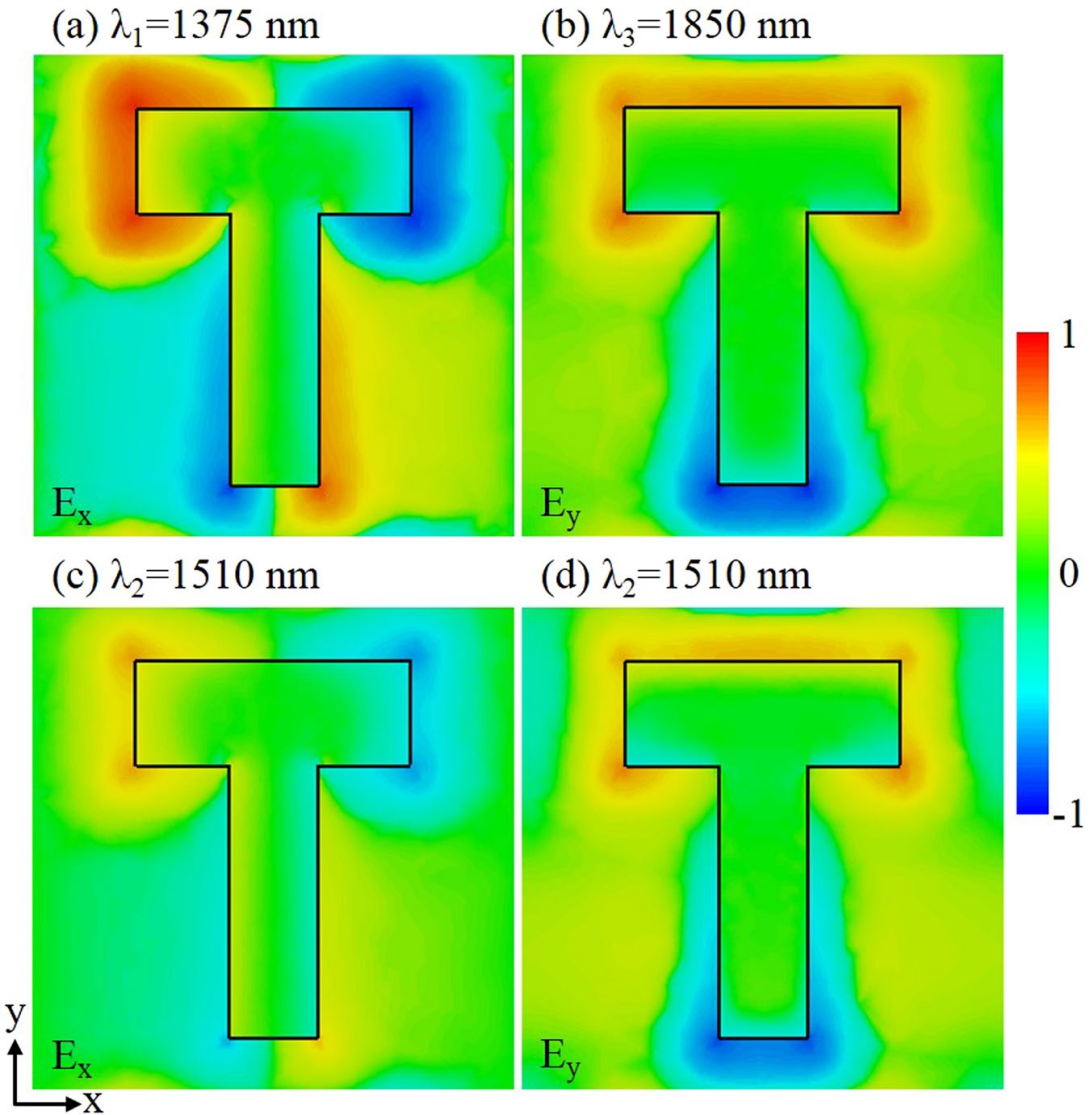
Simulated electric field distributions of plasmonic dipolar resonant modes supported in T-shaped antenna under linear polarization excitations at normal incidence (**a**) at the plasmonic resonance of horizontal arm *λ*_1_ = 1375 nm along the *x*-polarized direction, (**b**) at the plasmonic resonance of vertical arm *λ*_3_ = 1850 nm along the *y*-polarized direction, and (**c**), (**d**) at the crossing point of transmission amplitude spectra along both polarized directions.

Figure 4(a) plots the measured transmission amplitude spectra of *t*_xx_ and *t*_yy_ from the metasurface sample under two orthogonal linear polarization excitations along *x* and *y* directions, by using a Fourier-transform infrared (FTIR) spectrometer connected to an infrared microscope. It shows that two anisotropic plasmonic resonant modes are supported in the T-shaped antenna along its two arms with the resonance wavelengths located at *λ*_1_ = 1375 nm and *λ*_3_ = 1850 nm, respectively. The two plasmonic resonance dips cross at *λ*_2_ = 1510 nm where the transmission amplitudes of *t*_xx_ and *t*_yy_ are equal. It can be seen that the simulated transmission spectra agree with the measurement well. Figure 4(b) presents the measured and simulated phase retardation Δ*ϕ* between two orthogonal directions, as well as the simulated phase shifts *ϕ*_xx_ and *ϕ*_yy_ under two orthogonal linear polarizations. The measured phase retardation is between 78° and 82° which is very close to 90° for a perfect quarter-wave plate in a wide wavelength range from 1480 nm to 1580 nm, ensuring the functionality of high-performance broadband quarter-wave plate for our current metasurface design. The simulated phase retardation also matches the measurement well. In order to further understand the nature of anisotropic plasmonic resonances in the T-shaped antenna and elucidate the underlying mechanism of polarization conversion through the metasurface, the electric field distributions of T-shaped antenna at the two designed plasmonic resonance dips (*λ*_1_ = 1375 nm and *λ*_3_ = 1850 nm) and the crossing point of transmission amplitude spectra of *t*_*xx*_ and *t*_*yy*_ (*λ*_2_ = 1510 nm) are plotted in Fig. 5, under linear polarization excitations along *x* and *y* directions at normal incidence. It is shown in Fig. 5(a) and (b) that the resonant electric field is strongly concentrated at the ends of the antenna arm under linear polarization excitation, indicating a typical plasmonic dipolar resonance along each arm direction. As shown in Fig. 5(c) and (d), at the crossing point of transmission amplitude spectra, similar electric field distributions as those at the plasmonic resonance dips are obtained along both arm directions, but with weaker field intensity. In fact, the electric field distributions for the two plasmonic dipolar resonant modes shown in Fig. 5(c) and (d) exhibit a 90° phase difference so that nearly perfect linear-to-circular polarization conversion can be achieved.

According to ref. 23, in order to ensure that the metasurface works as an ideal ultrathin low-loss quarter-wave plate, the two nonzero diagonal terms of the transmission matrix should have the same amplitude, $|{t}_{xx}|\,=\,|{t}_{yy}|\,=\sqrt{2}/2$, and a phase difference exactly equal to 90°. In the case of ideal metal with infinite conductivity, the theoretical transmission coefficient is $\sqrt{2}/2$ for each orthogonal polarization component at the designed wavelength in perfectly lossless metasurfaces. In THz frequency range, most of metals can be nearly treated as perfectly electrical conducting materials, e.g. copper used in ref. 46. Thus, the transmission efficiencies along both x and y directions can be higher than 60%, close to the theoretical value, at the same time. However, metals such as gold become lossy in near-infrared wavelength range from 1480 nm to 1580 nm in our work. As a result, the transmission coefficient is significantly decreased. The transmission efficiency at the crossing point of transmission amplitude spectra shown in Fig. 4(a) is the optimized result that we can achieve under the current condition. The transmission efficiency could be further improved to approach the theoretical value by applying plasmonic materials with lower losses, together with advanced fabrication techniques.

The functionalities of our designed metasurface quarter-wave plate is further analyzed by using the degree of linear polarization (DoLP) and the angle of linear polarization (AoLP) for the output light wave according to the Stokes parameters^46–48^, which are the figure of merit to describe the efficiency of circular-to-linear polarization conversion. Stokes parameters are employed to calculate the output polarization states of light with four basic parameters related to the transmission amplitude *t*_xx_ and *t*_yy_ as well as the phase retardation Δ*ϕ*,

5 $${S}_{0}={|{t}_{xx}|}^{2}+{|{t}_{yy}|}^{2}$$

6 $${S}_{1}=\,{|{t}_{xx}|}^{2}-{|{t}_{yy}|}^{2}$$

7 $${S}_{2}=\,2|{t}_{xx}||{t}_{yy}|\cos \,{\rm{\Delta }}\varphi $$

8 $${S}_{3}=\,2|{t}_{xx}||{t}_{yy}|\sin \,{\rm{\Delta }}\varphi \,$$

The DoLP effectively represents how pure the transmitted linearly polarized light is for the incident circularly polarized light,

9 $$DoLP=\,\sqrt{{S}_{1}^{2}+{S}_{2}^{2}}/{S}_{0}$$

The AoLP is directly related to the specific rotation angle of the output linear polarization with respect to the *x*-axis,

10 $$AoLP=\arctan ({S}_{2}/{S}_{1})$$

A perfect quarter-wave plate has the DoLP of unity and the AoLP of 45°.

**Figure 6:**
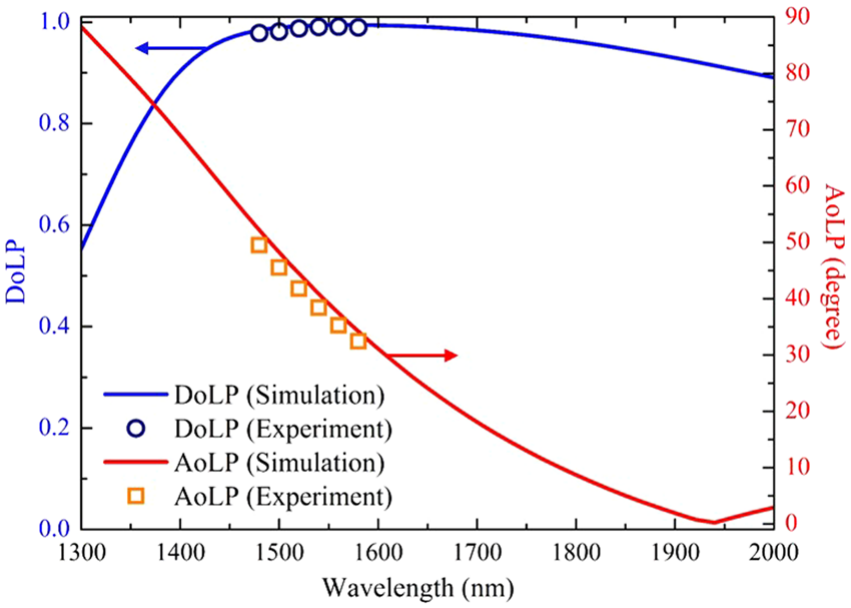
DoLP and AoLP extracted from experimental measurements and simulations for circularly polarized excitation at normal incidence.

Figure 6 plots the simulated and measured DoLP and AoLP for the transmitted light through the metasurface for a circularly polarized incident light. The measured DoLP in the broad bandwidth from 1480 nm to 1580 nm is greater than 0.98, which is consistent with the broadband phase retardation of nearly 90° as shown in Fig. 4(b), indicating nearly perfect linearly polarized light is obtained at the output. It is noted that the wavelength range for the simulated DoLP greater than 0.98 is from 1480 nm to 1720 nm covering a 240 nm bandwidth. It is observed that the AoLP is 45° as expected around the crossing point of transmission amplitude spectra at *λ* = 1510 nm. Due to the dispersion in the transmission amplitude spectra as shown in Fig. 4(a), the AoLP shifts in the range from 50° to 32° with respect to the *x*-axis between 1480 nm and 1580 nm in which the DoLP is nearly unity. The measured DoLP and AoLP are in good agreement with the simulation results. The weak dependence of AoLP on wavelength is usually desirable since it represents the fixed fast and slow axes from a point of view of a conventional quarter-wave plate. However, the fast and slow axes of the metasurface quarter-wave plate effectively rotate as a function of wavelength, which will have great potentials for sensing applications. According to the reciprocity theorem, it is expected that our demonstrated metasurface quarter-wave plate provides high-performance, broadband invertible polarization conversion between linearly and circularly polarized light.

## Discussion

In conclusion, we have demonstrated a high-performance broadband metasurface quarter-wave plate made of anisotropic T-shaped plasmonic antenna array at near-infrared wavelength. The transmission phase shifts induced by two anisotropic plasmonic dipolar resonances along the orthogonal arms of T-shaped antenna are directly measured by utilizing the optical vortex based interferometric approach. Extraordinary achromatic phase retardation up to 82° between two orthogonal directions with high transmission amplitude through the metasurface is achieved. The DoLP of near unity and the AoLP centered at 45° are obtained across a broad wavelength range for the metasurface quarter-wave plate, demonstrating high-performance broadband linear-to-circular polarization conversion. Our presented results will not only pave the way to the integration of metasurface based devices for polarization conversion, phase manipulation and structured light control in nanophotonic circuits and optical communication, but also open a new window for advancing optical spectroscopic and interferometric techniques to characterize metamaterials and metasurfaces.
